# Persistent neutrophil activation despite count normalization suggests immune dysregulation in exertional heat stroke

**DOI:** 10.3389/fimmu.2025.1718617

**Published:** 2026-02-04

**Authors:** Yue Wang, Siya Xu, Zhongzhi Tang, Jie Liu

**Affiliations:** 1 Anhui Institute of Optics and Fine Mechanics, Hefei Institutes of Physical Science, Chinese Academy of Sciences, Hefei, Anhui, China; 2 University of Science and Technology of China, Hefei, Anhui, China; 3 Department of Emergency, Central Theater General Hospital of The People’s Liberation Army of China, Wuhan, Hubei, China; 4 Department of Intensive Care Unit, The Affiliated Qingyuan Hospital (Qingyuan People’s Hospital), Guangzhou Medical University, Qingyuan, Guangdong, China

**Keywords:** exertional heat stroke, neutrophil activation, myeloperoxidase, neutrophil elastase, cytokines, longitudinal study

## Abstract

**Background:**

The role of neutrophils in exertional heat stroke (EHS), a life-threatening condition characterized by systemic inflammation, remains poorly defined. This study aimed to characterize the longitudinal profiles of neutrophil recruitment and activation in EHS patients.

**Methods:**

In this retrospective study with a small sample size, we analyzed clinical data and biobanked serum samples from 18 EHS patients and 18 matched healthy controls. Serum levels of interleukin-6 (IL-6), IL-8, IL-17, granulocyte colony-stimulating factor (G-CSF), CXCL1, CXCL2, C-reactive protein (CRP), serum amyloid A (SAA), myeloperoxidase (MPO), and neutrophil elastase (NE) were quantified by ELISA at onset and on days 3, 5, and 7 post-treatment.

**Results:**

At onset, EHS patients exhibited significant neutrophilia and elevated levels of IL-8, CXCL1, CXCL2, and IL-17 (all P < 0.05). While neutrophil counts normalized within days post-treatment, MPO and NE levels remained persistently and significantly elevated throughout the 7-day follow-up compared to controls (all P < 0.0001). Furthermore, strong positive correlations were observed between the levels of CXCL1/CXCL2 and MPO/NE at disease onset.

**Conclusion:**

In this exploratory study, our findings reveal a dissociation between neutrophil recruitment and activation in EHS. The persistent elevation of MPO and NE long after count normalization suggests a prolonged state of neutrophil dysregulation that could contribute to both acute tissue injury and long-term immune complications in EHS survivors.

## Introduction

Exertional heat stroke (EHS) is a life-threatening medical emergency characterized by central nervous system dysfunction and multi-organ failure (core temperature > 40 °C) that occurs during or following strenuous physical activity, often in hot and humid environments (1, 2). Its pathophysiology stems from a catastrophic failure of thermoregulation, where excessive heat production overwhelms the body’s capacity for heat dissipation, leading to a systemic inflammatory response syndrome (SIRS) that drives tissue injury (1, 3). As a significant risk to athletes, military personnel, and outdoor laborers, EHS represents a critical challenge in sports and occupational medicine (4).

A concerning sequela in heat stroke survivors is an acquired susceptibility to secondary infections, suggesting the presence of prolonged immune dysfunction that extends beyond the acute hyperinflammatory phase into a state of immunosuppression (5). For instance, in a cohort of heatstroke survivors from the 2003 French heat wave, a significant number of patients developed secondary infections during their hospital stay, contributing to poor outcomes (4). Similarly, long-term follow-up studies have indicated that EHS survivors can experience recurrent illnesses and altered immune responses for months after the initial insult (6, 7). This biphasic course—characterized by an acute SIRS often followed by a compensatory anti-inflammatory response syndrome (CARS) and prolonged immune alteration—highlights the complexity of EHS pathophysiology and underscores the need for research into the underlying immune mechanisms. While the innate immune system, particularly neutrophils as the first responders, is implicated in the initial hyperinflammatory state of EHS (8), their role may extend into a subsequent state of immunosuppression. Evidence supporting this includes observed neutrophilia coupled with impaired phagocytic function in heat stroke patients (9), suggesting a complex dysregulation of neutrophil biology.

Under physiological conditions, neutrophil recruitment and activation are tightly orchestrated processes. Egress from the bone marrow is primarily mediated by cytokines and chemokines such as granulocyte colony-stimulating factor (G-CSF), interleukin-17 (IL-17), C-X-C Motif Chemokine Ligand 1 (CXCL1), and C-X-C Motif Chemokine Ligand 2 (CXCL2) (which signal through C-X-C Motif Chemokine Receptor 2 [CXCR2]) (10–14). Subsequently, mediators like IL-8 guide neutrophils to sites of infection or injury, triggering effector functions that include the release of reactive oxygen species and proteolytic enzymes like myeloperoxidase (MPO) and neutrophil elastase (NE) to eliminate pathogens (15–17). However, when dysregulated, this powerful arsenal can cause significant bystander tissue damage, contributing to the pathology of sterile inflammatory diseases such as rheumatoid arthritis and ischemia-reperfusion injury (18, 19). In EHS, intense thermal stress is a potent trigger for such a dysregulated immune response, but the precise patterns of neutrophil activation and the cytokine milieu that drives it remain poorly defined.

Crucially, the longitudinal dynamics of neutrophil recruitment and function throughout the course of EHS, from the hyperacute onset to clinical recovery, have not been systematically investigated. It remains unknown whether the initial neutrophil activation resolves in parallel with clinical improvement or persists subclinically, potentially explaining the long-term vulnerability to infection. Therefore, to bridge this knowledge gap, we conducted a retrospective longitudinal study in patients with EHS. We aimed to comprehensively profile the temporal changes in 1) circulating neutrophil counts, 2) serum levels of key neutrophil-associated cytokines/chemokines (IL-8, CXCL1, CXCL2, IL-17, G-CSF), and 3) markers of neutrophil degranulation and activation (MPO and NE). We hypothesized that EHS would induce a distinct and persistent dysregulation of neutrophil biology, characterized by an early surge in both neutrophil numbers and activation markers that fails to resolve completely despite clinical recovery, thereby providing a potential mechanism for the immune dysfunction observed in these patients.

## Materials and methods

### Study design and population

This was a single-center, retrospective, observational study. We screened the clinical database and biobank of the Central Theater General Hospital for patients admitted with a diagnosis of EHS between June 2020 and October 2024. EHS was diagnosed according to the Chinese expert consensus on heat stroke (20). The diagnostic criteria required a history of strenuous exercise plus at least one of the following: 1) core temperature > 40 °C; 2) central nervous system (CNS) abnormalities (e.g., delirium, convulsions, or coma); 3) multiple organ (≥2) dysfunction; or 4) severe coagulopathy. Exclusion criteria were: classic (non-exertional) heat stroke, pre-existing neurological disorders, CNS infection, sepsis with an identifiable source, or age ≤ 18 years.

A total of 18 EHS patients who met the inclusion criteria and had available biobanked serum samples from at least two time points (including disease onset) were enrolled. The control group consisted of eighteen age- and sex-matched healthy volunteers. These control participants were recruited between 2021 and 2023, a period that overlapped with the patient enrollment phase, to minimize potential seasonal or environmental confounding. The single fasting serum samples collected from these controls were residual samples stored in the hospital’s biobank until analysis. All control participants had no history of recent infection, strenuous exercise, or chronic inflammatory conditions. Given the rarity of EHS and the practical challenges of longitudinal sample collection, the sample size was limited.

### Clinical management and data collection

Electronic medical records indicated that all enrolled EHS patients were managed according to a standardized institutional protocol, which included an emergent cooling strategy (20, 21) and comprehensive supportive care upon ICU admission. Clinical data extracted from the records included demographic characteristics, laboratory results, outcomes, and clinical scores (Glasgow Coma Scale [GCS], Sequential Organ Failure Assessment [SOFA], and Acute Physiology and Chronic Health Evaluation II [APACHE II]).

### Blood sample processing and biobanking

Peripheral blood samples (5 mL) were collected from EHS patients at several time points: upon hospital arrival (designated as onset) and on days 3, 5, and 7 after the initiation of treatment. For this study, these residual serum samples were retrieved from the hospital’s biobank. According to the biobank’s standard operating procedures, samples were processed by centrifugation at 3000 rpm for 10 minutes at room temperature to isolate serum, and aliquots were stored at -80°C. All samples were retrieved and analyzed in a single batch in early 2025. Prior to analysis, samples had been stored at -80°C for durations ranging from 3 months to 4.5 years. This single-batch analysis was conducted to avoid inter-assay variation.

The availability of samples varied across time points; not all patients had samples available for all four time points. Furthermore, the subset of patients with serum available for each specific biomarker assay varied due to differences in sample volume requirements. The specific sample size (n) for each analysis is provided in the corresponding figure legends and tables.

### Enzyme-linked immunosorbent assay

Serum concentrations of IL-6, C-reactive protein (CRP), serum amyloid A (SAA), IL-8, CXCL1, CXCL2, IL-17, G-CSF, MPO, and NE were quantified using commercial ELISA kits according to the manufacturers’ protocols. Kits for IL-6, CRP, SAA, CXCL1, CXCL2, MPO, NE, and G-CSF were from R&D Systems (Minneapolis, MN, USA); kits for IL-8 and IL-17 were from eBioscience (San Diego, CA, USA). The assay sensitivity for each analyte was as follows: IL-6 (9.4 pg/mL), CRP (15.6 ng/mL), SAA (1.6 ng/mL), IL-8 (2 pg/mL), CXCL1 (31.2 pg/mL), CXCL2 (15.6 pg/mL), G-CSF (9.4 pg/mL), MPO (4.62 pg/mL), NE (62.5 pg/mL). All samples were measured in duplicate. Due to limited residual sample volumes, total protein concentration was not measured; consequently, all ELISA results are reported as absolute concentrations in serum.

### Statistical analysis

Statistical analyses were performed using SPSS software (version 26.0; IBM Corp., Armonk, NY, USA). Categorical data are presented as numbers and frequencies (%), and continuous data are presented as median and interquartile range (IQR) due to their non-normal distribution, as assessed by the Shapiro-Wilk test. For baseline comparisons, differences between the EHS group at disease onset and the healthy control group were analyzed using the Mann-Whitney U test for continuous variables and the Chi-square or Fisher’s exact test for categorical variables. To evaluate longitudinal changes within the EHS cohort, the Kruskal-Wallis H test was employed as the primary method to assess overall differences in biomarker levels across all time points (onset, day 3, day 5, day 7), given the retrospective design and the resulting unbalanced data. If a significant overall difference was detected, *post-hoc* pairwise comparisons between each follow-up time point and the baseline (onset) were conducted using the Mann-Whitney U test, with a Bonferroni correction applied to adjust for multiple comparisons (significance level set at P < 0.0083). Furthermore, correlations between variables at disease onset were assessed using Spearman’s rank correlation coefficient. A two-sided P value of < 0.05 was considered statistically significant for all analyses except for the specified *post-hoc* tests. Non-parametric tests were selected as the most conservative and appropriate approach for the unbalanced data structure of this study.

## Results

### Baseline characteristics and clinical outcomes of EHS patients

A total of 18 patients with EHS and 18 healthy controls were enrolled. The baseline characteristics and clinical outcomes of the patients are summarized in **Table 1**. The median age of the EHS cohort was 26 years (IQR, 24-49), and 77.8% were male. All patients presented with a rectal temperature above 40°C (median, 42°C; IQR, 41.4–42.5°C) and exhibited signs of central nervous system injury (median GCS score, 5.5; IQR, 3.5–8.5). The median SOFA and APACHE II scores were 9.5 (IQR, 7–12.5) and 20.5 (IQR, 12.8–28.2), respectively, indicating significant multi-organ dysfunction. The median hospital length of stay was 7.6 days (IQR, 4.5–9.3 days). All patients survived without serious sequelae.

**Table 1:** Baseline characteristics and clinical outcomes of EHS patients and healthy controls.

Variable	Healthy controls (n=18)	EHS patients (n=18)	P value
Demographics			
Age, years, median (IQR)	30 (26–41)	26 (24–49)	0.601
Male sex, n (%)	12 (66.7)	14 (77.8)	0.711
Clinical presentation at admission			
Rectal temperature, °C, median (IQR)	36.5 (36.0-36.8)	42.0 (41.4-42.5)	<0.001***
Glasgow Coma Scale score, median (IQR)	15 (15–15)	5.5 (3.5-8.5)	<0.001***
SOFA score, median (IQR)	0 (0–0)	9.5 (7.0-12.5)	<0.001***
APACHE II score, median (IQR)	0 (0–0)	20.5 (12.8-28.2)	<0.001***
Clinical outcomes			
Hospital length of stay, days, median (IQR)	N/A	7.6 (4.5-9.3)	N/A

P-values were calculated using the Mann-Whitney U test for continuous variables and Chi-square or Fisher’s exact test for categorical variables. ***P < 0.001. EHS, Exertional Heat Stroke; IQR, Interquartile range; SOFA, Sequential Organ Failure Assessment; APACHE II, Acute Physiology and Chronic Health Evaluation II.

Consistent with the systemic inflammatory response in EHS, serum levels of acute-phase proteins, including CRP, SAA, and IL-6, were significantly elevated in patients compared to controls (all P < 0.05). Furthermore, markers of muscle injury (creatine kinase, myoglobin), liver dysfunction (alanine aminotransferase, aspartate aminotransferase, total and direct bilirubin), coagulation abnormalities (prothrombin time, activated partial thromboplastin time), and infection (procalcitonin) were all significantly higher in the EHS group (all P < 0.05) (**Table 2**).

**Table 2:** Laboratory parameters of EHS patients at disease onset and healthy controls.

Variable	Healthy controls (n=18), median (IQR)	EHS patients (n=18), median (IQR)	P value
Muscle Injury			
Mb, μg/L	53.2 (33.5-97.6)	135.7 (58.0-477.3)	0.014*
CK, IU/L	101.7 (86.6-138.7)	1201 (324.8-1942)	<0.001***
Liver function			
TBIL, μmol/L	11.70 (7.10-13.90)	17.60 (13.00-39.80)	0.001***
DBIL, μmol/L	3.50 (2.88-5.75)	6.70 (3.55-11.45)	0.020*
ALT, U/L	24.0 (17.8-34.7)	74.0 (26.0-357.5)	0.007**
AST, U/L	19.2 (16.1-27.4)	120.5 (38.8-446.0)	<0.001***
Coagulation			
PT, s	12.30 (11.43-12.93)	14.60 (12.30-16.20)	0.004**
APTT, s	24.25 (22.38-25.30)	33.50 (28.95-38.90)	<0.001***
Inflammation & infection			
PCT, ng/mL	0.025 (0.020-0.033)	0.560 (0.725-1.405)	<0.001***
CRP, mg/L	15 (8–34)	43 (6-27.9)	0.047*
SAA, mg/L	4.5 (1.3-5.3)	116.9 (16.5-457.3)	<0.001***
IL-6, pg/mL	0 (0–0)	4.28 (0-24.88)	0.001**
Other			
BUN, mmol/L	5.15 (4.38-5.98)	4.80 (4.15-7.15)	0.766
cTn-T, μg/L	0.0385 (0.0130-0.0538)	0.029 (0.011-0.081)	0.776

P-values were calculated using the Mann-Whitney U test. *P < 0.05, **P < 0.01, ***P < 0.001. EHS, Exertional Heat Stroke; IQR, Interquartile range; Mb, Myoglobin; CK, Creatine kinase; TBIL, Total bilirubin; DBIL, Direct bilirubin; ALT, Alanine aminotransferase; AST, Aspartate aminotransferase; PT, Prothrombin time; APTT, Activated partial thromboplastin time; PCT, Procalcitonin; CRP, C-reactive protein; SAA, Serum amyloid A; IL-6, Interleukin-6; BUN, Blood urea nitrogen; cTn-T, Cardiac troponin T.

### Neutrophil counts are transiently elevated in EHS patients

Hematological analysis revealed significant leukocytosis in EHS patients at onset, characterized primarily by neutrophilia. As detailed in **Table 3**, total leukocyte counts were significantly higher in EHS patients compared to controls [9.30 (IQR, 5.70–14.8) × 10³ vs. 6.60 (IQR, 5.45–8.35) × 10³ cells/µL; P = 0.012, r = 0.42]. This was driven by a marked increase in neutrophil counts [7.54 (IQR, 3.48–11.42) × 10³ vs. 4.22 (IQR, 2.30–5.77) × 10³ cells/µL; P = 0.001, r = 0.54]. The neutrophil-to-lymphocyte ratio was also significantly elevated in patients (6.59 [IQR, 2.32–9.68] vs. 2.47 [IQR, 1.67–3.43] in controls; P = 0.005, r = 0.47), while lymphocyte and monocyte counts showed no significant difference at onset (P = 0.058 and P = 0.939, respectively). Following treatment, leukocyte and neutrophil counts rapidly decreased to levels comparable to those of the control group at days 3, 5, and 7 (all P > 0.05). In contrast, lymphocyte counts in EHS patients demonstrated a delayed suppression, becoming significantly lower than those in controls by day 7 (P < 0.05) (**Table 3**).

**Table 3:** Longitudinal changes in leukocyte counts of EHS patients with available data during recovery.

Variable	Healthy controls (n=18)	EHS patients, median (IQR)				P value vs. control			
	median (IQR)	Onset (0d) (n=18)	Day 3 (n=8)	Day 5 (n=5)	Day 7 (n=7)	Onset (0d)	Day 3	Day 5	Day 7
Total leukocyte (×10³/µL)	6.60 (5.45-8.35)	9.30 (5.70-14.8)	6.95 (5.08-8.83)	5.20 (4.30-7.55)	4.80 (4.40-6.60)	<0.05*	0.785	0.465	0.211
Neutrophil (×10³/µL)	4.22 (2.30-5.77)	7.54 (3.48-11.42)	5.38 (2.75-7.74)	4.33 (1.84-6.37)	3.79 (2.15-5.54)	<0.05*	0.301	0.873	0.655
Lymphocyte (×10³/µL)	1.79 (1.32-2.45)	1.18 (0.59-2.12)	1.13 (0.60-1.88)	1.15 (0.52-1.95)	1.04 (0.52-1.88)	0.058	0.092	0.255	<0.05*
Monocyte (×10³/µL)	0.48 (0.39-0.72)	0.64 (0.24-0.80)	0.35 (0.25-0.53)	0.35 (0.27-0.60)	0.46 (0.26-0.70)	0.939	0.185	0.465	0.754
Neutrophil-to-Lymphocyte Ratio	2.47 (1.67-3.43)	6.59 (2.32-9.68)	4.84 (1.34-12.29)	5.34 (1.13-11.22)	3.75 (1.03-10.65)	<0.05*	0.104	0.065	0.327

P-values indicate significant differences compared to the healthy control group for each respective time point using the Mann-Whitney U test. ***P < 0.001. EHS, Exertional Heat Stroke; IQR, Interquartile range.

### Elevated levels of neutrophil-associated cytokines and chemokines at EHS onset

**Figure 1:**
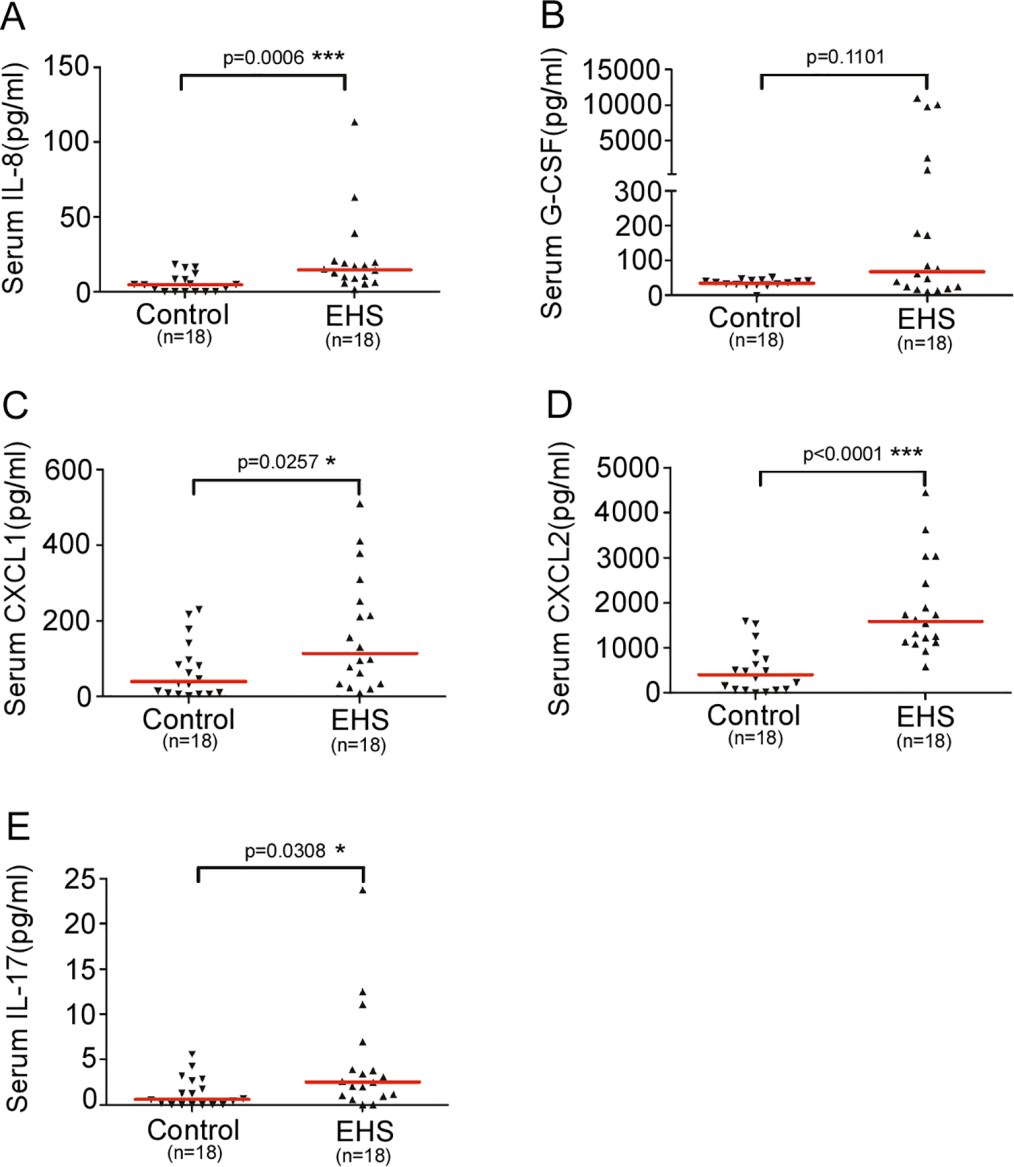
Levels of serum IL-8, G-CSF, CXCL1, CXCL2 and IL-17 in patients with EHS. Scatter plot shows the levels of serum IL-8 **(A)**, G-CSF **(B)**, CXCL1 **(C)**, CXCL2 **(D)** and IL-17 **(E)** in the control (n=18) and EHS (n=18) groups. Horizontal red bars represent the median for each group. *P < 0.05, ***P < 0.001. IL-8, Interleukin-8; G-CSF, Granulocyte Colony-Stimulating Factor; CXCL1, C-X-C Motif Chemokine Ligand 1; CXCL2, C-X-C Motif Chemokine Ligand 2; IL-17, Interleukin-17; EHS, Exertional Heat Stroke.

To profile the inflammatory milieu, we measured serum levels of cytokines and chemokines implicated in neutrophil recruitment and function. At disease onset, EHS patients (n=18) exhibited significantly elevated levels of IL-8, CXCL1, CXCL2, and IL-17 compared to healthy controls, as shown in **Figures 1A, C–E**. Specifically, IL-8 levels were 14.57 (IQR, 8.12–19.69) pg/mL in patients versus 4.70 (IQR, 0–9.40) pg/mL in controls (P = 0.0006). CXCL1 levels were 113.9 (IQR, 33.04–266.2) pg/mL versus 39.4 (IQR, 8.17–108.0) pg/mL (P = 0.0257), and IL-17 levels were 2.53 (IQR, 1.01–4.66) pg/mL versus 0.611 (IQR, 0–2.71) pg/mL (P = 0.0308). CXCL2 levels were also significantly higher in patients (P < 0.0001). In contrast, G-CSF levels did not differ significantly between the two groups (P = 0.1101; **Figure 1B**).

**Figure 2:**
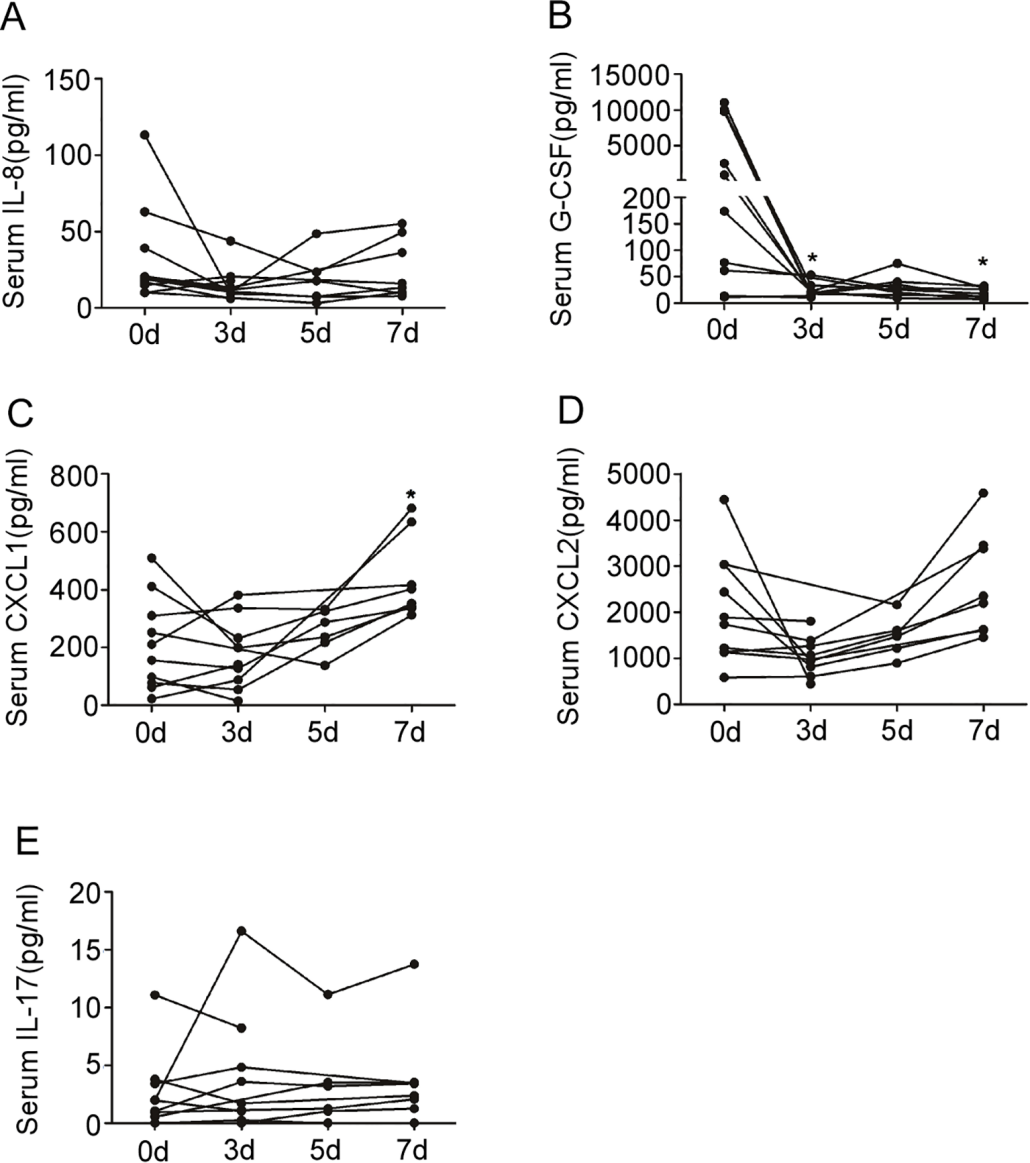
Changes in serum IL-8, G-CSF, CXCL1, CXCL2 and IL-17 in patients with EHS after treatment. **(A)** Serum levels of IL-8 in EHS patients at onset (n=10), 3 days (n=9), 5 days (n=6) and 7 days (n=8) after treatment. **(B)** Serum levels of G-CSF in EHS patients at onset (n=10), 3 days (n=9), 5 days (n=6) and 7 days (n=8) after treatment. **(C)** Serum levels of CXCL1 in EHS patients at onset (n=10), 3 days (n=9), 5 days (n=6) and 7 days (n=8) after treatment. **(D)** Serum levels of CXCL2 in EHS patients at onset (n=10), 3 days (n=9), 5 days (n=6) and 7 days (n=8) after treatment. **(E)** Serum levels of IL-17 in EHS patients at onset (n=10), 3 days (n=9), 5 days (n=6) and 7 days (n=8) after treatment. *P < 0.05 compared to the level at onset (Kruskal-Wallis test with *post-hoc* analysis). IL-8, Interleukin-8; G-CSF, Granulocyte Colony-Stimulating Factor; CXCL1, C-X-C Motif Chemokine Ligand 1; CXCL2, C-X-C Motif Chemokine Ligand 2; IL-17, Interleukin-17; EHS, Exertional Heat Stroke. Sample sizes vary across time points due to the retrospective study design and sample availability. The cohort of patients with serum available for cytokine analysis differed from the overall cohort presented in **Table 3**. The specific ‘n’ for each time point is indicated in the figure.

The longitudinal dynamics of these mediators were then assessed during the recovery phase (**Figure 2**). Longitudinal analysis revealed a significant overall difference in G-CSF levels (P < 0.05), with *post-hoc* tests showing significant decreases at days 3 and 5 compared to onset (both P < 0.05; **Figure 2B**). In contrast, no significant overall differences were detected for IL-8 (**Figure 2A**) or IL-17 (**Figure 2E**). Intriguingly, a significant overall difference was found for CXCL1 (P < 0.05), driven by a significant increase by day 7 after treatment (P < 0.05; **Figure 2C**). No such late increase was observed for CXCL2 (**Figure 2D**).

### Persistent elevation of neutrophil degranulation markers despite count normalization

**Figure 3:**
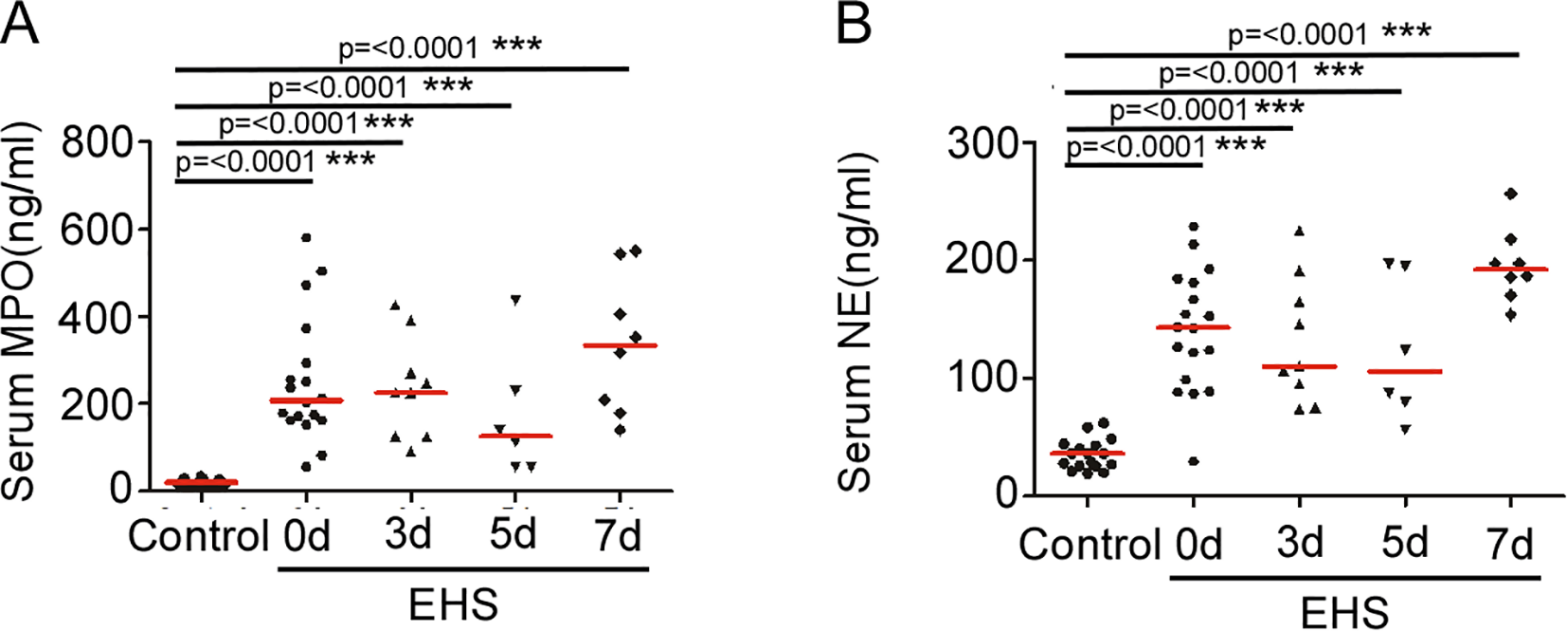
Levels of serum MPO and NE in controls and patients with EHS after treatment. **(A)** Scatter plots showing serum levels of MPO in the control (n=18) and EHS groups at onset (n=18), 3 days (n=9), 5 days (n=6) and 7 days (n=8) after treatment. **(B)** Scatter plots showing serum levels of NE in the control (n=18) and EHS groups at onset (n=18), 3 days (n=9), 5 days (n=6) and 7 days (n=8) after treatment. Horizontal red bars represent the median for each group. ***P < 0.001. MPO, Myeloperoxidase; NE, Neutrophil Elastase; EHS, Exertional Heat Stroke. Sample sizes vary across time points due to the retrospective study design and sample availability. The specific ‘n’ for each group and time point is indicated in the figure or its description.

To assess neutrophil activation, we measured serum levels of the primary granule components MPO and NE. At onset, MPO and NE levels were profoundly elevated in EHS patients compared to controls (MPO: 208.6 [IQR, 163.1–313.3] ng/mL vs. 20.88 [IQR, 13.6–26.12] ng/mL, P < 0.0001, r = 0.89; NE: 143.0 [IQR, 96.43–182.0] ng/mL vs. 36.49 [IQR, 25.90–43.76] ng/mL, P < 0.0001, r = 0.85). More importantly, a striking dissociation was observed: despite the subsequent normalization of neutrophil counts, serum MPO and NE levels remained persistently elevated throughout the 7-day follow-up (**Figures 3A, B**). Levels at all post-treatment time points were significantly higher than those in controls (all P < 0.0001) but did not differ significantly from levels at onset (all P > 0.05).

### Correlation between neutrophil chemoattractants and activation markers

**Figure 4:**
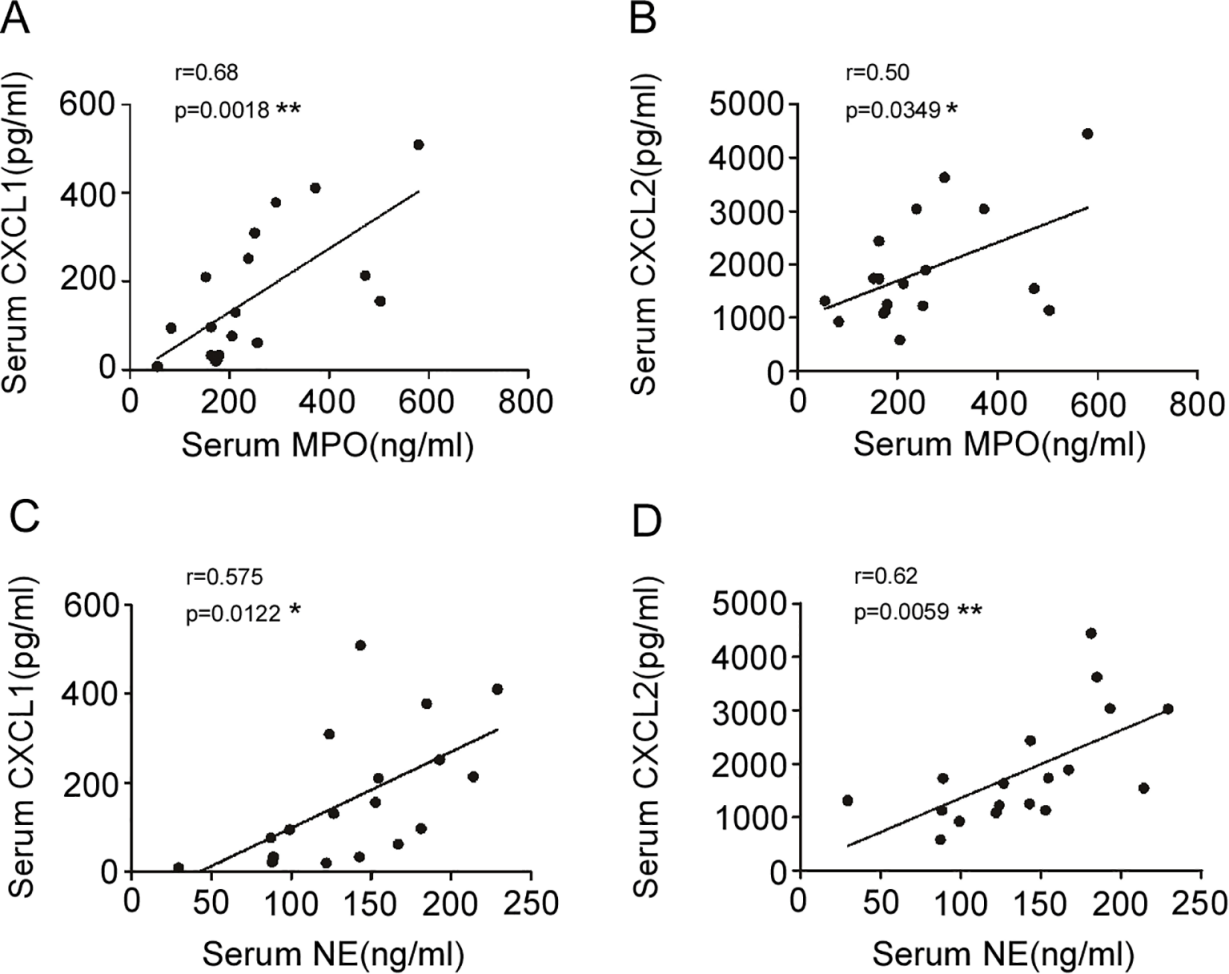
The association of serum CXCL1 and CXCL2 with serum MPO and NE in EHS patients at onset (Spearman’s correlation analysis). **(A)** Correlation between serum CXCL1 and serum MPO (n=18); **(B)** Correlation between serum CXCL2 and serum MPO (n=18). **(C)** The correlation between serum CXCL1 and serum NE (n=18). **(D)** Correlation between serum CXCL2 and serum NE (n=18). *P < 0.05, **P < 0.01. CXCL1, C-X-C Motif Chemokine Ligand 1; CXCL2, C-X-C Motif Chemokine Ligand 2; MPO, Myeloperoxidase; NE, Neutrophil Elastase; EHS, Exertional Heat Stroke. Sample sizes vary across time points due to the retrospective study design and sample availability. The specific ‘n’ for each group and time point is indicated in the figure or its description.

We performed correlation analysis to explore potential relationships between neutrophil chemoattractants and markers of degranulation. Serum levels of both CXCL1 and CXCL2 at onset showed significant positive correlations with levels of MPO (r = 0.68, 95% CI [0.32, 0.86], P = 0.0018, **Figure 4A**; r = 0.50, 95% CI [0.03, 0.78], P = 0.0349, **Figure 4B**, respectively) and NE (r = 0.575, 95% CI [0.15, 0.82], P = 0.0122, **Figure 4C**; r = 0.62, 95% CI [0.22, 0.84], P = 0.0059, **Figure 4D**, respectively). No significant correlations were observed between other cytokines (IL-8, IL-17, G-CSF) and MPO or NE levels (data not shown).

## Discussion

In the present study, we demonstrate a pronounced dissociation between neutrophil abundance and activation state in patients with EHS. We observed that blood neutrophil counts increased significantly at onset but normalized rapidly with treatment, a finding consistent with a previous report (22). Concurrently, levels of key neutrophil-recruiting cytokines/chemokines (IL-8, IL-17, CXCL1, and CXCL2) were markedly elevated, suggesting that the initial neutrophilia is likely driven by a cytokine storm that promotes bone marrow egress and recruitment to tissues.

The most salient and novel finding of our study, however, was the distinct longitudinal dynamic. Despite the rapid resolution of neutrophilia, MPO and NE remained significantly elevated throughout the 7-day observation period, even after circulating neutrophil counts had normalized. This discordant pattern indicates that EHS induces a profound and persistent dysregulation of neutrophil function that outlasts the quantitative abnormality. This functional alteration could involve the release of immature neutrophil subsets with altered activity profiles (23) or delayed apoptosis (24); both mechanisms warrant further investigation in EHS.

It is important to consider alternative explanations for the persistent elevation of MPO and NE, which might not arise solely from ongoing neutrophil activation. Potential mechanisms include delayed clearance of these enzymes from the circulation, continued release from pre-formed pools within damaged tissues, or impaired enzymatic degradation. Although our current data cannot definitively distinguish between these possibilities, the strong correlation between CXCL1/CXCL2 levels (potent activators of degranulation) and MPO/NE levels at disease onset supports the concept of active, chemokine-driven release. This correlation specifically points to a role for the CXCR2 signaling pathway, given that CXCL1 and CXCL2 are its potent agonists and this receptor is known to promote neutrophil degranulation upon activation (25). This interpretation is consistent with studies demonstrating that CXCR2 antagonism can reduce MPO activity (26). Furthermore, the significant elevation of serum levels throughout the 7-day follow-up period, a time during which clinical recovery was underway, suggests a sustained process rather than a consequence of a single initial insult. Future studies incorporating functional assays of neutrophil activity (e.g., phagocytosis, oxidative burst) and direct measurements of clearance pathways are needed to resolve the precise mechanisms underlying the persistent neutrophil dysregulation in EHS.

We hypothesize that during EHS, a massive SIRS triggers both neutrophilia and robust neutrophil activation, leading to tissue infiltration and secondary organ damage (27–30). The persistence of these proteolytic enzymes in the circulation, even during clinical recovery, suggests a prolonged state of immune dysregulation. This lingering subclinical inflammation may contribute to the long-term immune dysfunction observed in EHS survivors, including an elevated risk of infection (4, 31, 32). The persistent elevation of proteolytic and pro-inflammatory mediators like MPO and NE, even after count normalization, may have direct clinical implications. Given that MPO and NE are known to cause bystander tissue damage and disrupt endothelial integrity (6, 7), their sustained elevation could contribute to the delayed organ injury observed in some heat stroke survivors. Moreover, our observation of persistent neutrophil dysregulation aligns with the established phenomenon of immunosuppression following other critical illnesses, such as sepsis (33). In these conditions, a maladaptive immune response often features both impaired lymphocyte function and dysregulated neutrophil activity, culminating in an increased susceptibility to secondary infections (34). The parallel between these well-characterized immunoparalytic states and our findings provides a plausible immunological mechanism for the clinical observation of acquired infection susceptibility post-EHS (4) and suggests that EHS may instigate a similar long-term immunodysregulation. While this mechanistic link is compelling, it is important to note that our study was not powered to directly correlate biomarker levels with specific clinical outcomes like infection rates. Therefore, establishing a definitive causal relationship between persistent neutrophil activation and long-term clinical complications remains a critical avenue for future research with dedicated long-term follow-up. Our findings are supported by studies showing altered heat shock protein responsiveness in leukocytes months after an episode (35) and persistent epigenetic reprogramming of immune cells following EHS (36).

We acknowledge several limitations in our study. The statistical power and generalizability are constrained by the retrospective, single-center design, small sample size (n=18), and sample attrition over time. Furthermore, without an exercise-heat control group, we cannot definitively isolate the pathology of EHS from the physiological effects of exertion. This is an inherent constraint of the retrospective design, which precluded the prospective enrollment of such a control group. Perhaps most importantly, while we document a prolonged state of neutrophil dysregulation, our study design does not allow us to link this finding to specific long-term clinical outcomes, such as infection risk—a critical question for future research. The predominantly male cohort also limits insights into sex-specific responses. Thus, while our work provides the first longitudinal evidence of persistent neutrophil activation in human EHS, revealing a potential mechanism for post-EHS immune complications, these exploratory findings must be validated in larger, prospective, multi-center studies that include diverse participants, appropriate control groups, and clinical outcome data.

## Conclusion

In conclusion, our study reveals a dissociation between neutrophil recruitment and activation in EHS. The persistent elevation of MPO and NE indicates a long-lasting dysregulation of neutrophil biology, potentially driven by CXCL1/CXCL2 signaling. Future prospective, multi-center studies with larger cohorts are necessary to validate these findings. Furthermore, functional assays on neutrophils from EHS patients are required to substantiate this immune dysregulation hypothesis and to explore the underlying cellular mechanisms. Ultimately, understanding these processes may reveal novel therapeutic targets, such as strategies to modulate neutrophil activation or promote its resolution, for the benefit of EHS survivors.

## Data Availability

The original contributions presented in the study are included in the article/supplementary material. Further inquiries can be directed to the corresponding authors.

## References

[1] Gaudio FG Grissom CK . Cooling methods in heat stroke. J Emerg Med. (2016) 50:607–16. doi: 10.1016/j.jemermed.2015.09.014, PMID: 26525947

[2] Bouchama A Knochel JP . Heat stroke. N Engl J Med. (2002) 346:1978–88. doi: 10.1056/NEJMra011089, PMID: 12075060

[3] Leon LR Bouchama A . Heat stroke. Compr Physiol. (2015) 5:611–47. doi: 10.1002/j.2040-4603.2015.tb00612.x 25880507

[4] Argaud L Ferry T Le QH Marfisi A Ciorba D Achache P . Short- and long-term outcomes of heatstroke following the 2003 heat wave in Lyon, France. Arch Intern Med. (2007) 167:2177–83. doi: 10.1001/archinte.167.20.ioi70147, PMID: 17698677

[5] Walsh NP Whitham M . Exercising in environmental extremes: a greater threat to immune function? Sports Med. (2006) 36:941–76. doi: 10.2165/00007256-200636110-00003, PMID: 17052132

[6] Aratani Y . Myeloperoxidase: Its role for host defense, inflammation, and neutrophil function. Arch Biochem Biophys. (2018) 640:47–52. doi: 10.1016/j.abb.2018.01.004, PMID: 29336940

[7] Pham CT . Neutrophil serine proteases: specific regulators of inflammation. Nat Rev Immunol. (2006) 6:541–50. doi: 10.1038/nri1841, PMID: 16799473

[8] Dale DC Boxer L Liles WC . The phagocytes: neutrophils and monocytes. Blood. (2008) 112:935–45. doi: 10.1182/blood-2007-12-077917, PMID: 18684880

[9] Lacy P . Mechanisms of degranulation in neutrophils. Allergy Asthma Clin Immunol. (2006) 2:98–108. doi: 10.1186/1710-1492-2-3-98, PMID: 20525154 PMC2876182

[10] Segal AW . How neutrophils kill microbes. Annu Rev Immunol. (2005) 23:197–223. doi: 10.1146/annurev.immunol.23.021704.115653, PMID: 15771570 PMC2092448

[11] Sheshachalam A Srivastava N Mitchell T Lacy P Eitzen G . Granule protein processing and regulated secretion in neutrophils. Front Immunol. (2014) 5:448. doi: 10.3389/fimmu.2014.00448, PMID: 25285096 PMC4168738

[12] Cecchi I Arias de la Rosa I Menegatti E Roccatello D Collantes-Estevez E Lopez-Pedrera C . Neutrophils: Novel key players in Rheumatoid Arthritis. Current and future therapeutic targets. Autoimmun Rev. (2018) 17:1138–49. doi: 10.1016/j.autrev.2018.06.006, PMID: 30217550

[13] Anzai A Shimoda M Endo J Kohno T Katsumata Y Matsuhashi T . Adventitial CXCL1/G-CSF expression in response to acute aortic dissection triggers local neutrophil recruitment and activation leading to aortic rupture. Circ Res. (2015) 116:612–23. doi: 10.1161/CIRCRESAHA.116.304918, PMID: 25563839

[14] Zhang Z Yuan W Deng J Wang D Zhang T Peng L . Granulocyte colony stimulating factor (G-CSF) regulates neutrophils infiltration and periodontal tissue destruction in an experimental periodontitis. Mol Immunol. (2020) 117:110–21. doi: 10.1016/j.molimm.2019.11.003, PMID: 31765840

[15] Ray A Kolls JK . Neutrophilic inflammation in asthma and association with disease severity. Trends Immunol. (2017) 38:942–54. doi: 10.1016/j.it.2017.07.003, PMID: 28784414 PMC5711587

[16] Eash KJ Greenbaum AM Gopalan PK Link DC . CXCR2 and CXCR4 antagonistically regulate neutrophil trafficking from murine bone marrow. J Clin Invest. (2010) 120:2423–31. doi: 10.1172/JCI41649, PMID: 20516641 PMC2898597

[17] Tyrkalska SD Candel S Angosto D Gomez-Abellan V Martin-Sanchez F Garcia-Moreno D . Neutrophils mediate Salmonella Typhimurium clearance through the GBP4 inflammasome-dependent production of prostaglandins. Nat Commun. (2016) 7:12077. doi: 10.1038/ncomms12077, PMID: 27363812 PMC4932187

[18] Liew PX Kubes P . The neutrophil’s role during health and disease. Physiol Rev. (2019) 99:1223–48. doi: 10.1152/physrev.00012.2018, PMID: 30758246

[19] Kuwabara T Ishikawa F Kondo M Kakiuchi T . The role of IL-17 and related cytokines in inflammatory autoimmune diseases. Mediators Inflamm. (2017) 2017:3908061. doi: 10.1155/2017/3908061, PMID: 28316374 PMC5337858

[20] Liu SY Song JC Mao HD Zhao JB Song Q Expert Group of Heat Stroke P . Expert consensus on the diagnosis and treatment of heat stroke in China. Mil Med Res. (2020) 7:1. doi: 10.1186/s40779-019-0229-2, PMID: 31928528 PMC6956553

[21] Casa DJ McDermott BP Lee EC Yeargin SW Armstrong LE Maresh CM . Cold water immersion: the gold standard for exertional heatstroke treatment. Exerc Sport Sci Rev. (2007) 35:141–9. doi: 10.1097/jes.0b013e3180a02bec, PMID: 17620933

[22] Lu KC Lin SH Chu P Tsai WS Lin YF . Correlation of neutrophil phagocytosis and lymphocyte adhesion molecules in exertional heat stroke. Am J Med Sci. (2004) 327:68–72. doi: 10.1097/00000441-200402000-00002, PMID: 14770021

[23] Pillay J Ramakers BP Kamp VM Loi AL Lam SW Hietbrink F . Functional heterogeneity and differential priming of circulating neutrophils in human experimental endotoxemia. J Leukoc Biol. (2010) 88:211–20. doi: 10.1189/jlb.1209793, PMID: 20400675

[24] Gupta S Lee CM Wang JF Parodo J Jia SH Hu J . Heat-shock protein-90 prolongs septic neutrophil survival by protecting c-Src kinase and caspase-8 from proteasomal degradation. J Leukoc Biol. (2018) 103:933–44. doi: 10.1002/JLB.4A0816-354R, PMID: 29393970

[25] Hipkin RW Deno G Fine J Sun Y Wilburn B Fan X . Cloning and pharmacological characterization of CXCR1 and CXCR2 from Macaca fascicularis. J Pharmacol Exp Ther. (2004) 310:291–300. doi: 10.1124/jpet.103.063131, PMID: 15028780

[26] Bento AF Leite DF Claudino RF Hara DB Leal PC Calixto JB . The selective nonpeptide CXCR2 antagonist SB225002 ameliorates acute experimental colitis in mice. J Leukoc Biol. (2008) 84:1213–21. doi: 10.1189/jlb.0408231, PMID: 18653784

[27] Yang HH Hou CC Lin MT Chang CP . Attenuating heat-induced acute lung inflammation and injury by dextromethorphan in rats. Am J Respir Cell Mol Biol. (2012) 46:407–13. doi: 10.1165/rcmb.2011-0226OC, PMID: 22033269

[28] Zhou G Chen Z Li J Guo X Qin K Luo J . Role of the receptor for advanced glycation end products in heat stress-induced endothelial hyperpermeability in acute lung injury. Front Physiol. (2020) 11:1087. doi: 10.3389/fphys.2020.01087, PMID: 33192536 PMC7643755

[29] Hu J Kang H Liu C Hu P Yang M Zhou F . Regulatory T cells could improve intestinal barrier dysfunction in heatstroke. Inflammation. (2019) 42:1228–38. doi: 10.1007/s10753-019-00983-6, PMID: 30820807

[30] Peng N Geng Y Zhang S Tang Y Wen Q Tong H . Correlation of kidney injury and inflammatory response in rats with classic severe heatstroke. Zhonghua Wei Zhong Bing Ji Jiu Yi Xue. (2015) 27:327–31. doi: 10.3760/cma.j.issn.2095-4352.2015.05.002, PMID: 26003634

[31] Davido A Patzak A Dart T Sadier MP Meraud P Masmoudi R . Risk factors for heat related death during the August 2003 heat wave in Paris, France, in patients evaluated at the emergency department of the Hopital Europeen Georges Pompidou. Emerg Med J. (2006) 23:515–8. doi: 10.1136/emj.2005.028290, PMID: 16794091 PMC2579541

[32] Dematte JE O’Mara K Buescher J Whitney CG Forsythe S McNamee T . Near-fatal heat stroke during the 1995 heat wave in Chicago. Ann Intern Med. (1998) 129:173–81. doi: 10.7326/0003-4819-129-3-199808010-00001, PMID: 9696724

[33] Hotchkiss RS Monneret G Payen D . Sepsis-induced immunosuppression: from cellular dysfunctions to immunotherapy. Nat Rev Immunol. (2013) 13:862–74. doi: 10.1038/nri3552, PMID: 24232462 PMC4077177

[34] Alves-Filho JC de Freitas A Spiller F Souto FO Cunha FQ . The role of neutrophils in severe sepsis. Shock. (2008) 30 Suppl 1:3–9. doi: 10.1097/SHK.0b013e3181818466, PMID: 18704017

[35] Ruell PA Simar D Periard JD Best S Caillaud C Thompson MW . Plasma and lymphocyte Hsp72 responses to exercise in athletes with prior exertional heat illness. Amino Acids. (2014) 46:1491–9. doi: 10.1007/s00726-014-1721-3, PMID: 24633453

[36] Murray KO Brant JO Iwaniec JD Sheikh LH de Carvalho L Garcia CK . Exertional heat stroke leads to concurrent long-term epigenetic memory, immunosuppression and altered heat shock response in female mice. J Physiol. (2021) 599:119–41. doi: 10.1113/JP280518, PMID: 33037634

